# Isolation, Characterization and Bioactivities of an Extracellular Polysaccharide Produced from *Streptomyces* sp. MOE6

**DOI:** 10.3390/molecules22091396

**Published:** 2017-08-24

**Authors:** Marwa O. Elnahas, Magdy A. Amin, Mohamed M. D. Hussein, Vinit C. Shanbhag, Amal E. Ali, Judy D. Wall

**Affiliations:** 1 Department of Biochemistry, University of Missouri, Columbia, MO 65211, USA; elnahasm@missouri.edu (M.O.E.); vcs36d@mail.missouri.edu (V.C.S.); 2 Department of Microbiology and Immunology, Faculty of Pharmacy, Cairo University, Cairo 11562, Egypt; magdy_biotek3@yahoo.com (M.A.A.); amal.ali@pharma.cu.edu.eg (A.E.A.); 3 Department of Chemistry of Natural and Microbial Products, National Research Center, Cairo 12622, Egypt; dr_mmagdeldin@yahoo.com

**Keywords:** *Streptomyces*, polysaccharides, antioxidant activities, tumor inhibition

## Abstract

A *Streptomyces* strain was isolated from soil and the sequence of 1471 nucleotides of its 16S rDNA showed 99% identity to *Streptomyces* sp. HV10. This newly isolated *Streptomyces* strain produced an extracellular polysaccharide (EPS) composed mainly of glucose and mannose in a ratio of 1:4.1, as was characterized by Fourier transform infrared spectroscopy (FTIR), HPLC and ^1^H-NMR. The antioxidant activities of the partially purified MOE6-EPS were determined by measuring the hydroxyl free radical scavenging activity and the scavenging of 2,2-diphenyl-2-picryl-hydrazyl (DPPH) radicals. In addition, the partially purified MOE6-EPS showed high ferrous ion (Fe^2+^) chelation activity which is another antioxidant activity. Interestingly, 3-(4,5-dimethylthiazol-2-yl)-2,5-diphenyltetrazolium bromide (MTT) assays that were colorimetric assays for NAD(P)H-dependent cellular oxidoreductases and a proxy of the number of viable cells, showed that the partially purified MOE6-EPS inhibited the proliferation of the human breast cancer cells (MDA-MB-231). The scratch wound assay showed that MOE6-EPS reduced the migration of mouse breast cancer cells (4T1). This study reports the production of EPS from *Streptomyces* species with promising antioxidant, metal chelating and mammalian cell inhibitory activities.

## 1. Introduction

Microbial polysaccharides are high molecular weight polymers consisting of carbohydrates [1]. These polysaccharides exist in different forms: covalently bound to the cell surface as capsular polysaccharides or secreted into the surrounding environment as free exopolysaccharides (EPS). Microbial polysaccharides have different biological functions: protecting the cell from desiccation, acting as antimicrobial agents, facilitating the adhesion of bacteria to various solid surfaces, and playing an important role in symbiosis [2,3]. EPS may be homopolysaccharides, which are composed of a single type of monosaccharide forming the polysaccharide chain, or heteropolysaccharides, which are chains composed of two or more different sugars usually with different ratios [4]. The different structures and saccharide composition of EPS produced from different microbial strains hold the potential for diverse functionalities.

Microbial polysaccharides are used extensively in various industrial and pharmacological applications due to their unique physical and rheological properties. These polymers can be used as gelling, emulsifying, thickening or flocculating agents in food, cosmetics and adhesive industries, as well as waste water treatment and oil recovery applications [5]. Interestingly, certain EPS products have demonstrated functional biological activities when used as antitumor, antiviral, anticoagulant and/or immunomodulating agents [6,7].

The role of Reactive Oxygen Species (ROS) is well established in the development of many diseases including cancer, cellular aging and neurodegeneration, as well as coronary heart disease, because ROS oxidatively damage tissues and cellular machinery [8,9]. Since many of the synthetic antioxidants have unfavorable side effects [10], the development of treatments has focused on the production of antioxidant drugs of natural origin. Several microbial polysaccharides have been reported to have strong antioxidant activities and may be developed as natural antioxidant drugs [11,12].

While the production of microbial EPS from bacteria is extensively studied, the understanding of its production from *Streptomyces* species is incomplete. *Streptomyces* species are Gram positive, filamentous bacteria found mainly in soil and they are well known producers of biologically active compounds, mainly antibiotics [13]. In this study, we report on a novel isolate from the soil of Columbia, MO, USA with 16S rDNA sequence homologous to *Streptomyces* sp. HV10. The newly isolated strain produces EPS, that shows apparent antioxidant, metal chelating activities, as well as inhibition of proliferation and migration of mammalian cells.

## 2. Results and Discussion

### 2.1. Isolation and Identification of the Streptomycetes

**Figure 1:**
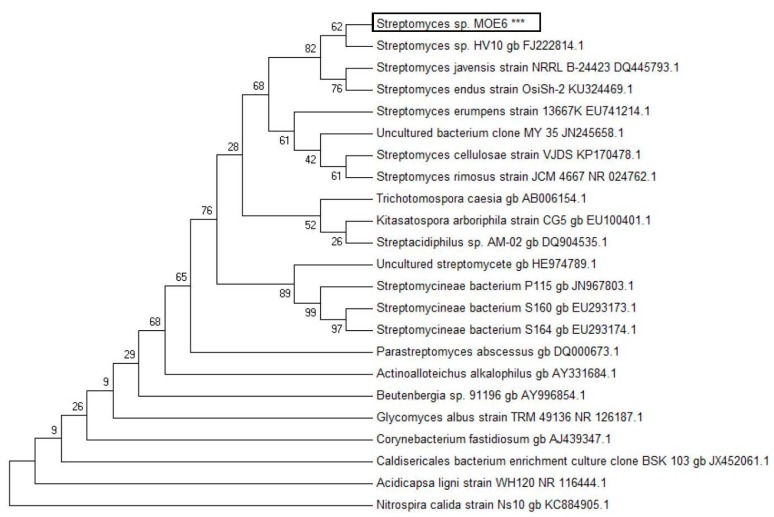
Maximum likelihood phylogenetic tree of the isolated strain *Streptomyces* sp. MOE6 based on 16S rRNA gene sequencing of a 1471 bp fragment. *** indicates the MOE6 isolate.

A Gram-positive, filamentous *Streptomyces* species was isolated from soil sample of Columbia, Missouri, USA; the colonies are white, chalky and circular shaped, which is characteristic of the Streptomycetaceae family. A 1471-base pair fragment of the 16S rDNA of the isolate was sequenced, and the genus of the organism was assigned with a maximum likelihood phylogenetic tree and sequence alignment (Figure 1). Based on high (>99%) sequence identity of the 16S rDNA gene to that of *Streptomyces* sp. HV10 and position on the *Streptomyces* branch of the phylogenetic tree, the isolate was assigned to the *Streptomyces* genus. The 16S rDNA sequence has been submitted to GenBank (accession number KY742742, available at https://www.ncbi.nlm.nih.gov/genbank/).

### 2.2. Characterization of Streptomyces sp. MOE6 Exopolysaccharide

Following extraction, partial purification and lyophilization of the EPS, the final product deemed MOE6-EPS had a yield of (2.5 g/L) and was observed to be a white, spongy precipitate. MOE6-EPS was then subjected to structural and molecular content characterization by spectroscopy and sugar analyses to confirm the product to be EPS and to understand its physical properties. 

**Figure 2:**
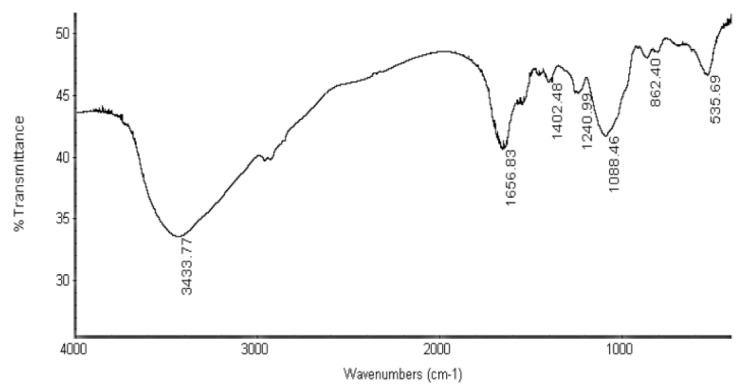
Fourier transform infrared (FTIR) spectrum of the MOE6-EPS in the range of 400–4000 cm^−1^ showing common bands which are characteristic for polysaccharides.

Fourier transform infrared (FTIR) spectroscopy was employed to detect the main functional groups in MOE6-EPS [14]. The FTIR spectrum (Figure 2) showed that the MOE6-EPS contains the typical absorption peaks associated with polysaccharides. The broad, large peak at 3433 cm^−1^ indicates the presence of hydroxyl groups which are characteristic for polysaccharides, as each monosaccharide has more than one hydroxyl [15]. The bands in the regions of 2962 cm^−1^ and 1200 cm^−1^ to 1400 cm^−1^ are assigned to weak CH-stretching of methylene (–CH2–) groups and angular vibration which is evidence that the produced MOE6-EPS contains carbohydrate [16,17]. Moreover, the band around 1656 cm^−1^ may result from C=O stretch of the carboxylic group for uronic acid [18]. In the spectrum, the absorption at 1088 cm^−1^ region is attributed to the stretching vibration of pyranose ring [19]. Taken together, all of these features in the FTIR spectrum confirm that the product is a polysaccharide containing pyranoses.

**Figure 3:**
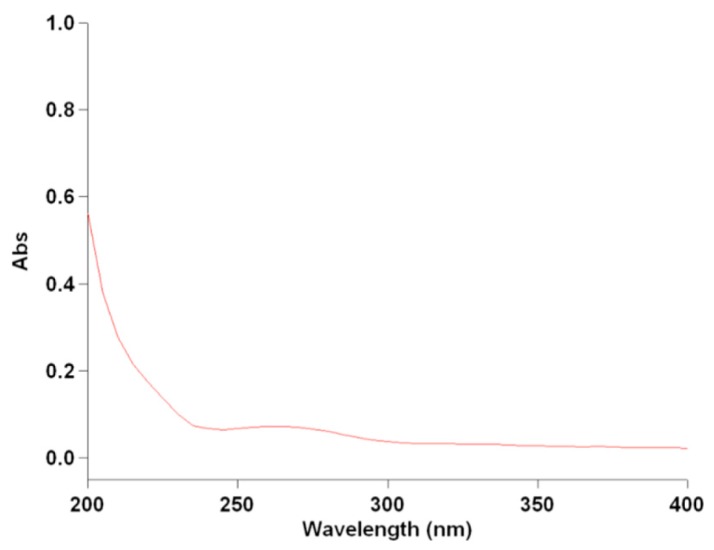
UV-visible absorption spectrum for MOE6-EPS in the range of (200–400 nm).

Additional spectroscopic characterization of the partially purified MOE6-EPS was carried out with UV-visible absorption spectroscopy (Figure 3). The absorption spectrum of 250 μg MOE6-EPS/mL had maximum absorption in the range of 200–210 nm, with a small shoulder in the range of 250–290 nm. The region between 200–210 nm could be attributed to the presence of carboxyl, carbonyl, or ester functional groups, while the presence of bands at 250–290 nm is commonly due to the presence of aromatic compounds. The bands at 260 nm and 280 nm may be attributed to DNA and protein respectively [20]. The UV-vis spectrum provided further evidence that the MOE6-EPS product could be composed of carbohydrates. Since spectroscopic studies were suggestive of sugar content found in polysaccharides, MOE6-EPS was tested chemically for carbohydrate content and the sugars typically found in MOE6-EPS. The phenol sulfuric assay [21] showed that the partially purified MOE6-EPS contains 71 ± 1% carbohydrate. The metahydroxydiphenyl assay for the uronic acids [22] revealed that the MOE6-EPS contains 3.6 ± 0.1% uronic acid while the Bradford assay for protein content [23] showed that MOE6-EPS contains 0.78 ± 0.1%.

**Figure 4:**
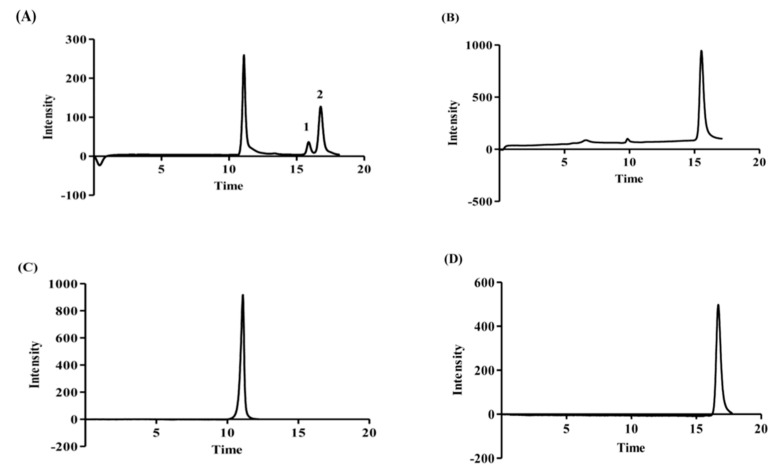
HPLC chromatograph of (**A**) hydrolyzed MOE6-EPS (10 mg/mL) from isolate MOE6 strain; (**B**) glucose control (5 mg/mL); (**C**) glucuronic acid control (5 mg/mL); (**D**) mannose control (5 mg/mL).

The monosaccharide units of the MOE6-EPS were then identified by HPLC retention times compared to the retention times of reference sugars including: glucose, galactose, mannose, xylose and glucuronic acid. After hydrolysis with 2M trifluoroacetic acid (TFA) at 110 °C for 6 h, the sugars were analyzed on the HPLC and the refractive index chromatograms of each sugar were recorded. In the neutral sugar region, two distinct peaks (Figure 4, peaks 1 and 2) on the MOE6-EPS chromatogram were observed at retention times corresponding to those of glucose (15.9 min) and mannose (16.7 min) and were assigned peaks 1 and 2, respectively (Figure 4). By comparing peak areas of the two sugars, the ratio of glucose to mannose in MOE6-EPS was calculated to be 1:4.1, respectively. Similarly, Miyazaki and Yamada [24] reported that *Streptomyces* sp. FERM-P1185, which also was isolated from soil, produces an EPS which is composed of repeating units of glucose and mannose in molar ratio 1.87:1. This comparison suggests that even bacteria from the same genus produce EPS with unique sugar content. The polysaccharide may also contain non-carbohydrate moieties such as acetate and pyruvate as in the case of xanthan gum which contains 85.3% carbohydrates formed mainly of glucose, mannose and glucuronic acid together with acetate and pyruvate, the concentrations of the acetate and pyruvate varied depending on the fermentation process conditions [25]. The reference sugars were run on HPLC before and after hydrolysis, and it was found that after hydrolysis, an additional peak appeared at a retention time, which is so close to that of the glucuronic acid, and it overlapped the glucuronic acid peak making an identification and quantification of glucuronic acid indeterminate.

**Figure 5:**
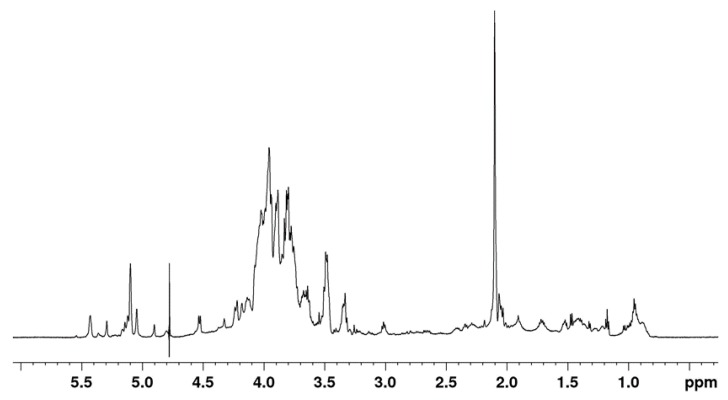
Proton NMR spectrum of 5 mg of MOE6-EPS dissolved in 0.75 mL D_2_O.

The partially purified MOE6-EPS was further characterized by one-dimensional (1D) ^1^H-NMR spectroscopy (Figure 5). The ^1^H-NMR spectrum of polysaccharides consists mainly of three regions. The first region is the ring proton region (δH 3.1–4.5 ppm) which shows a crowded signal region due to the presence of many sugar residues and this region is typical for polysaccharides. The second region is the anomeric proton region (δH 4.5–5.5 ppm) and, finally, the third region is the alkyl region (δH 1.2–2.3 ppm) [26]. The ^1^H-NMR spectrum of MOE6-EPS showed the ring proton region which is characteristic of polysaccharides, and it also showed a signal at δ 2.102 ppm indicating that the polysaccharide contains acetyl groups. Moreover, the ^1^H-NMR spectrum of MOE6-EPS contained six main anomeric protons. The anomeric region of the NMR spectrum revealed the presence of α-anomeric and β-anomeric protons, due the presence of signals between δ 4.90 ppm to δ 5.50 ppm and δ 4.30 ppm to δ 4.80 ppm, respectively. The proton signals at 5.04 ppm and 4.9 ppm may be attributed to the presence of the hydrogen (H1) that is binding to the carbon (C1) of both mannose and glucose respectively. These results are similar to what was shown by ^1^H-NMR spectroscopy of a glucomannan isolated from *Amorphophallus* species that had signals at 4.9 ppm and 5.05 ppm due to the presence of H1 of glucose and mannose units, respectively [27]. The ^1^H-NMR spectrum confirms the results obtained from HPLC which showed that the MOE6-EPS contains glucose and mannose.

**Figure 6:**
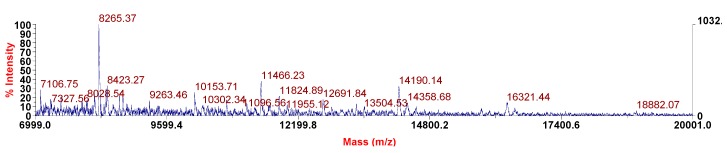
MALDI-TOF of MOE6-EPS.

The matrix-assisted laser desorption ionization-time of flight (MALDI-TOF) analysis of MOE6-EPS (Figure 6) showed the presence of 4 prominent peaks at 8265 (±2 to 5 Da; as determined by the University of Missouri Charles W. Gehrke Proteomics Center), 11,465; 14,190; and 16,320 Da suggesting that MOE6-EPS may be a low molecular weight polysaccharide. However, it has not been established whether the original product is actually much larger or whether it could not be ionized for analysis. If the product were large, then the fragments identified could have resulted from damage occurring during the purification process or the laser ablation. 

We found additional evidence in the spectrum that the EPS is most likely a polysaccharide. The mass differences between the base peak at 8423 and its closest fragment peak at 8265 is approximately 158 Da, suggesting a six-carbon sugar monomer (180 less water + proton = 163 Da) was lost from the chain. Also a similar mass difference, 169, was found between peak at 14,359 and 14,190. Therefore, the molecular mass of the polymer is at least 8 to 16 kDa.

### 2.3. Antioxidant Activities of MOE6-EPS

EPS products isolated from many bacteria have demonstrated antioxidant activity by scavenging free radicals. This is a desired reaction in many biological systems to prevent ROS induced oxidative damage. The efficiency of this reaction depends on the sugar content and structure of each EPS [28]. Since EPS products from different bacteria have unique compositions, the ability of each strain’s EPS to act as an antioxidant will be characteristic of the composition. ROS includes radical and non-radical oxygen species which are generated from the partial reduction of oxygen. When the production of these radicals increases, they will be out of control of the cellular antioxidant defense system generating an oxidative stress [29]. This oxidative stress results in the damage of many biologically active molecules including nucleic acids, lipids, and proteins that contribute to the development of several diseases in mammals, such as neurodegenerative diseases [30] and cancer [29]. Hence, it is crucial to develop novel antioxidant compounds particularly of natural sources to avoid possible side effects of synthetic antioxidants. 

#### 2.3.1. 2,2-Diphenyl-1-picrylhydrazyl (DPPH) Radical Scavenging

**Figure 7:**
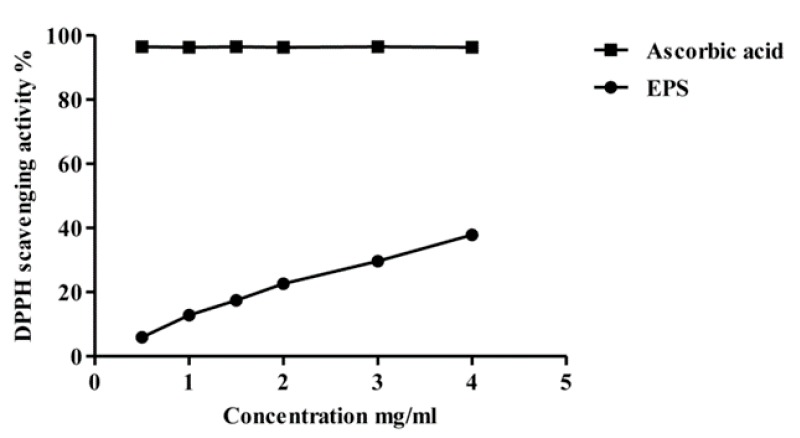
2,2-Diphenyl-1-picrylhydrazyl (DPPH) radicals scavenging activity of different concentrations of MOE6-EPS (0.5, 1, 1.5, 2, 3, 4 mg/mL) from *Streptomyces* sp. MOE6 compared to that of ascorbic acid (0.5, 1, 1.5, 2, 3, 4 mg/mL) as a positive control. Values are means ± SD (*n* = 3). 100% (*w/v*) of DPPH was 78.86 μg/mL. The absence of error bars indicates that the errors were smaller than the symbols.

The DPPH assay has been extensively used to evaluate the ability of different compounds to scavenge free radicals. The scavenging of free radicals is one mechanism of antioxidant activity. When the unpaired electron on the nitrogen atom of DPPH receives a hydrogen atom from antioxidants, it will be reduced and lose its deep purple color and form a pale yellow color [31]. The decrease in the absorbance at 517 nm is used to measure the DPPH radical scavenging activity of the tested compound. Figure 7 shows the DPPH radical scavenging activity of the partially purified MOE6-EPS compared to ascorbic acid, a very strong antioxidant. The ascorbic acid was used as a positive control and it showed higher DPPH scavenging activity at the same weights used for EPS. The DPPH scavenging activity of MOE6-EPS was concentration dependent and linearly increased over the MOE6-EPS concentrations tested. At 0.5 mg/mL, the EPS showed scavenging ability of 6.0 ± 0.1% of the DPPH free radicals, while at 4 mg/mL, the EPS showed 37.8 ± 0.1% scavenging of initial DPPH concentration.

#### 2.3.2. Hydroxyl Radical Scavenging Activity

A second antioxidant activity assay measures the ability of the partially purified MOE6-EPS to scavenge hydroxyl radicals. The hydroxyl radical •OH is a potent reactive oxygen species formed by the partial reduction of oxygen [32]. 

The antioxidant capacity of the purified MOE6-EPS was tested as described by Yin et al., (2010) with the phenanthroline assay. In this assay, the hydroxyl radicals are generated through the following reaction:

Fe^2+^ + H_2_O_2_ → Fe^3+^ + OH^−^ + •OH,

In this reaction, there are two mechanisms for the antioxidation activity. The first mechanism is the direct scavenging of the formed hydroxyl radicals to form a non-radical compound. And the second mechanism is through the suppression of the formation of these hydroxyl radicals by chelating the Fe^2+^ required for the generation of hydroxyl radicals. These iron complexes are rendered unable to react with H_2_O_2_ to form •OH [33]. Therefore, the polysaccharides with metal chelating activities should be able to reduce the generation of •OH through the chelation Fe^2+^ and thus display antioxidant activity. The phenanthroline assay does not discriminate between these two mechanisms and the scavenging ability seen in this assay can result from direct reduction of hydroxyl radicals, the suppression of radical generation by metal chelation or a combination of both mechanisms.

**Figure 8:**
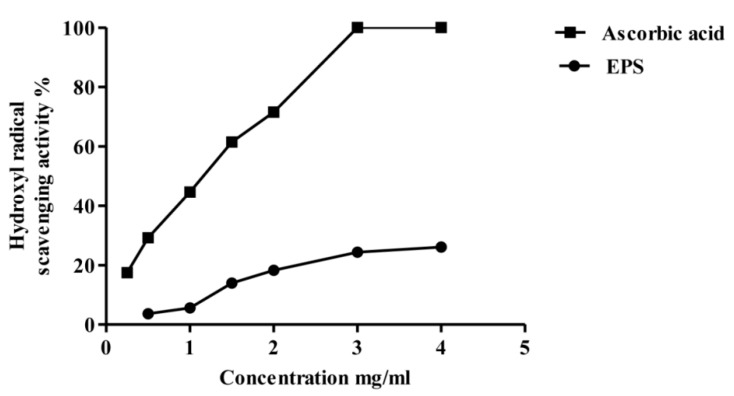
Hydroxyl radical scavenging activity of different concentrations of MOE6-EPS (0.5, 1, 1.5, 2, 3, 4 mg/mL) from *Streptomyces* sp. MOE6 compared to that of ascorbic acid (0.25, 0.5, 1, 1.5, 2, 3, 4 mg/mL) as a positive control. Values are means ± SD (*n* = 3). 100% *w/v* of OH^−^ was 340 μg /mL. The absence of error bars indicates that the errors were smaller than the symbols.

Figure 8 shows the phenanthroline assay of the partially purified MOE6-EPS hydroxyl radical scavenging activity compared to ascorbic acid as a positive control. The hydroxyl radical scavenging activity increased with increasing concentrations of MOE6-EPS in the range of 0.5–4 mg/mL. At 0.5 mg/mL, MOE6-EPS showed hydroxyl radical scavenging activity of 3.6 ± 0.1% while the maximum hydroxyl radical scavenging activity measured was 26.0 ± 0.1% achieved at 4 mg/mL EPS. By contrast, at 4 mg/mL ascorbic acid had nearly 100% hydroxyl radical scavenging activity. Like the MOE6-EPS, the ascorbic acid scavenging activity increased with increasing its concentration until, unlike the MOE6-EPS, ascorbic acid reached a maximum scavenging activity (100%) at 3 mg/mL with no further increase in the activity observed above this concentration. The low scavenging activity of the EPS could be attributed to the metal chelating activity of the polymer. To decipher if MOE6-EPS is acting as an antioxidant through direct reduction or metal chelation, Fe^2+^ chelating activity of the EPS in a ferrozine-based assay was performed.

#### 2.3.3. Ferrous Ion Chelating Activity of Purified EPS

**Figure 9:**
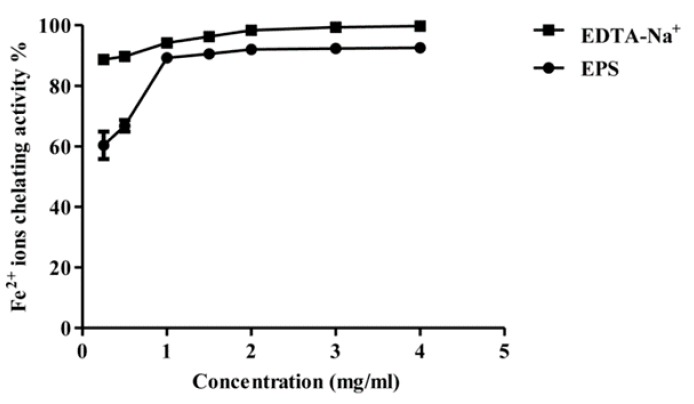
Ferrous ion chelating activity of different concentrations of MOE6-EPS (0.25, 0.5, 1, 1.5, 2, 3, 4 mg/mL) from *Streptomyces* sp. MOE6 compared to that of EDTA-Na^+^ (0.25, 0.5, 1, 1.5, 2, 3, 4 mg/mL) as a positive control. Values are means ± SD (*n* = 3). 100% (*w/v*) Fe^2+^ was 111.6 μg/mL. The absence of error bars indicates that the errors were smaller than the symbols.

Ferrous ion chelation (Fe^2+^) activity is strongly related to the antioxidant activity, due to the ability of transition metal ions to initiate radical formation through the transfer of a single electron to certain compounds thereby generating free radicals [34,35]. Metal chelating activity of the partially purified MOE6-EPS was measured following the method reported by Qiao and co-workers [34,36], where the absorbance at 562 nm of the red colored complex of ferrozine with reduced iron was measured. The addition of a metal-chelating agent disrupts the formation of the ferrozine–Fe^2+^ complex decreasing the red color formed by the complex. In Figure 9, MOE6-EPS showed strong ferrous ion chelation activity which increased with increasing concentrations of the MOE6-EPS as compared to EDTA–Na^+^ at similar EPS concentrations. The MOE6-EPS maximum chelating capacity occurred at a concentration of 2 mg/mL which was 92.0 ± 0.1% where EDTA–Na^+^ had a 98.4 ± 0.02% chelating capacity at the same concentration (2 mg/mL). There was no apparent increase in the metal chelation activity upon increasing the EPS concentration beyond 2 mg/mL. The chelating ability of a compound can be described as the formation of bonds between two or more binding sites within the same molecule with one single central atom [37]. Since the characterization results showed that the produced MOE6-EPS contains uronic acid, the negatively charged carboxyl groups could act as the binding sites for ferrous ions [38,39]. Similar results were obtained by Li et al., 2014 [40] who reported three EPS products isolated from *Lactobacillus helveticus* MB2-1. All three of the EPS products had the ability to chelate ferrous ion under the same experimental conditions, and the metal chelating activities of these polysaccharides increased by increasing the polysaccharide concentrations reaching maximum activity at 2 mg/mL as observed for MOE6-EPS. Additionally, as seen for MOE6-EPS, a further increase in the polysaccharide concentration above 2 mg/mL did not show any significant effect on the ferrous iron chelating activity. However, MOE6-EPS had almost twice the binding capacity of the *Lactobacillus* EPS products. The three EPS products of *Lactobacillus* showed metal chelating activities of 53.2 ± 0.1%, 45.9 ± 0.1 and 38.7 ± 0.1% each at a concentration of 4 mg/mL. These results suggest that MOE6-EPS is a strong metal chelator.

There are several factors that affect the antioxidant activities of the polysaccharides. You et al., 2013 [41] showed that the antioxidant activity of the polysaccharide was affected by the molecular weight, the smaller the molecular weight the higher the antioxidant activity. Based on this finding, the difference in scavenging ability of the MOE6-EPS as compared to ascorbic acid could be attributed to the difference in molecular weight, as the MOE6-EPS in the assay was not hydrolyzed. Structural studies of MOE6-EPS would help resolve the mechanisms of radical scavenging and metal chelation. Furthermore, the monosaccharide composition and the structure of the polymer also have been shown to affect the free radical scavenging activities [28]. In future studies, the structure of the MOE6-EPS will be determined allowing interpretation of the structure–activity relationship of the MOE6-EPS in antioxidant activity mechanisms. Taken together, the sugar-composition characterization and antioxidant assays suggest that MOE6-EPS might prove useful as an antioxidant and metal-chelator.

### 2.4. Effect of MOE6-EPS on the Migration and Proliferation of Mammalian Cells

Understanding the effects of *Streptomyces* EPS on mammalian cell growth may provide the basis for the development of a product useful for intervention in disease. Breast cancer is the most frequently diagnosed cancer type and is one of the leading causes of cancer death worldwide [42]. ROS levels are elevated in all cancer types as they modulate the behavior of cancer cells and are involved in cell proliferation, cell apoptosis, angiogenesis, metabolism, protein synthesis and inflammation [43].

Additionally, the essential contribution of iron to tumor growth is well established [44]. Iron is crucial for the activity of iron-and heme-containing enzymes that are involved in the mitochondrial activity, DNA synthesis and cell cycle [45]; hence, iron supports tumor growth through promoting cell replication and metabolism [46,47]. Also, iron induces the generation of deleterious hydroxyl radical ROS that results in DNA damage and the generation of carcinogenic mutations. Interestingly, several cancer types, including breast cancer, show elevated iron acquisition and storage due to the increased expression levels and activity of the proteins responsible for iron metabolism in malignant cells, e.g., transferrin, lipocalin and ferritin [46,48,49,50,51]. As such, therapeutic approaches targeting iron metabolism focus on iron chelators [52,53] where some have shown promise in preclinical and clinical studies as anticancer agents [53,54,55]. Based on our results with scavenging ROS and chelation of iron, we sought to evaluate the inhibitory activity of the partially purified EPS of isolate MOE6 toward the murine (4T1) and human breast cancer cells (MDA-MB 231) as a first step in determining the effectiveness of MOE6-EPS as an inhibitor of mammalian cell growth and development.

#### 2.4.1. The Scratch Wound Assay

**Figure 10:**
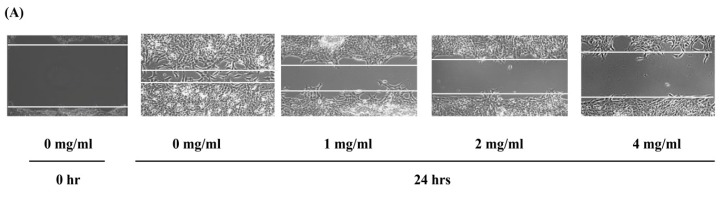

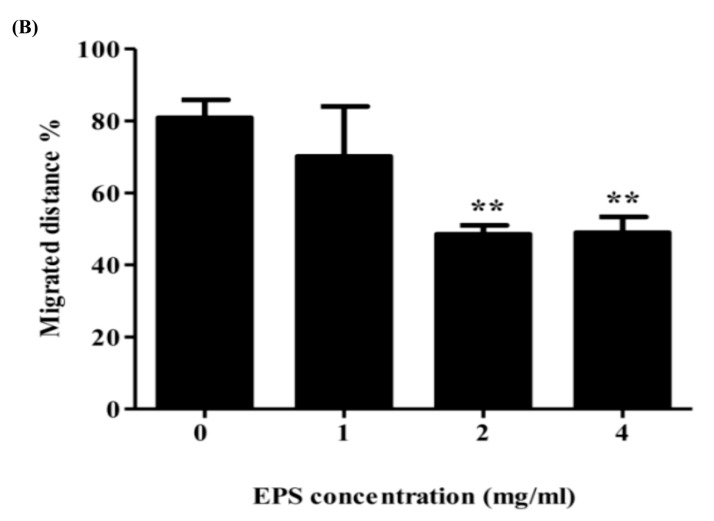
MOE6-EPS reduces the migration of 4T1 murine breast cancer cells. The 4T1 cells were cultured in 0, 1, 2 and 4 mg MOE6-EPS/mL. The wound healing was monitored for 24 h by real time microscopy and images were captured after 0 and 24 h. (**A**) Representative images of 3 independent experiments; (**B**) Quantification and statistical analysis of the migrated distance by 4T1 cells as a percentage of control. Values are means ± SD (*n* = 3) where ** *p* < 0.01 indicate significant decreases from untreated cells.

Often cancer involves uncontrolled cell migration that is the first step in metastasis [43], the process by which cancer cells migrate away from their original tumor site and disseminate throughout the body [44]. The migration ability of tumor cells is crucial for the metastasis of various cancer types including breast cancer cells [56,57]. As a result, a scratch wound assay was used to assess the influence of the EPS on the migration [58] of murine breast cancer cells (4T1 cells). In the scratch assay, an artificial gap (scratch) is created after the cancer cells form a confluent monolayer. The cells at the edge of the scratch will migrate closing this scratch and forming a monolayer once more [58]. Here, the migration of 4T1 cells was monitored by real time microscopy through imaging at regular intervals following the addition of different concentrations of the partially purified MOE6-EPS. The results revealed that the MOE6-EPS treatment significantly reduced the migration ability of murine breast cancer 4T1 cells in a concentration dependent manner. The percentage of migration distance was 80.9% in untreated cells, and was significantly decreased upon treatment with EPS to 70.1%, 48.5% and 48.9% at concentrations of 1, 2 and 4 mg/mL respectively (Figure 10). This result demonstrates that the EPS inhibits murine breast cancer cell migration.

#### 2.4.2. MTT Assay

**Figure 11:**
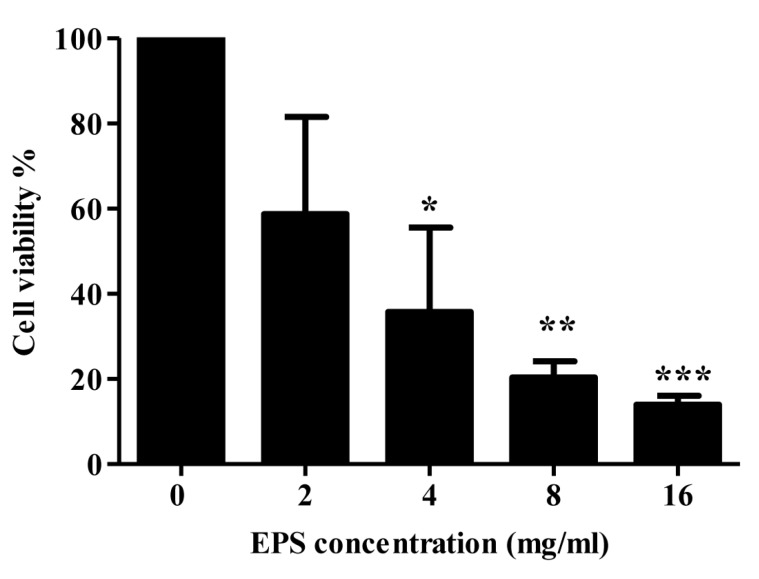
MOE6-EPS reduces the proliferation of MDA-MB 231 human breast cancer cells. MDA-MB 231 cells were cultured in 0, 2, 4, 8 and 16 mg MOE6-EPS/mL for 96 h and then the cell viability was assessed by the MTT assay. Values are means ± SD (*n* = 3), where * *p* < 0.05, ** *p* < 0.01 and *** *p* < 0.001 indicate significant decreases from untreated cells.

Another major therapeutic approach for cancer therapy is to inhibit uncontrolled cancer cell proliferation, a typical characteristic of various cancer types [59]. The anti-proliferative activity of the partially purified MOE6-EPS was assessed by the MTT assay, where the yellow MTT will be reduced to purple formazan by cellular mitochondrial dehydrogenase found in the living cells [60], thus the increase in staining of viable human breast cancer cells MDA-MB 231 will be proportional to the living cells [61]. The MTT assay showed that the EPS has a significant effect on the proliferation of MDA-MB 231 breast cancer cells, where the cell viability decreased with the increase of the MOE6-EPS concentrations and this growth inhibitory activity is dose-dependent at EPS concentrations ranging from (0–16 mg/mL) (Figure 11). The IC_50_ of EPS was calculated to be 2.93 mg/mL. Compared with the negative control (untreated), the partially purified MOE6-EPS showed significant inhibition (*p* < 0.05) and (*p* < 0.01) at 4 mg/mL and 8–16 mg/mL respectively, which as mentioned earlier, might be attributed to the iron chelating of the EPS. Further investigation on the mechanism of the inhibitory activity of the EPS in a variety of tumor-bearing and non-tumor cell lines will be pursued in our future work. Thus, we report for the first time that the *Streptomyces* sp. MOE6 produces EPS with apparent activity against the proliferation of human breast cancer, one of the most prevalent cancer types and with the highest mortality rate among women [42]. Further studies will determine whether the EPS inhibition occurs differentially with normal cells being more resistant than cancer cells, a desired result for the development of a useful treatment product.

## 3. Materials and Methods

### 3.1. Materials and Reagents

2,2-Diphenyl-1-picrylhydrazyl (DPPH), trifluoroacetic acid (TFA), EDTA-Na, KBr, FeCl_2_·4H_2_O_,_ MgSO_4_·7H_2_O, anhydrous CaCO_3_, ZnSO_4_·7H_2_O, galactose, mannose, xylose were purchased from Sigma Chemical Co. (St. Louis, MO, USA). 1,10-phenanthroline, m-hydroxybiphenyl, H_2_O_2,_ phenol, H_2_SO_4_, yeast extract, tryptone, glucose, agar, NaCl, glycerol, anhydrous K_2_HPO_4_, KH_2_PO_4_, ascorbic acid were purchased from Fisher Scientific Co. (Fair Lawn, NJ, USA). Ethanol was purchased from Decon Labs, Inc. (King of Prussia, PA, USA). Ferrozine was purchased from HACH Chemical Co. (Ames, IA, USA). Malt was purchased from MP Biomedicals, LLC. (Solon, OH, USA). Dulbecco’s Modified Eagle Medium (DMEM) was purchased from Thermo Fisher Scientific (Waltham, MA, USA).

Cell cultures: the mouse breast cancer cell line (4T1 cells ATCC CRL-2539) and the human breast cancer cell line (MDA-MB-231 ATCC HTB-26) were purchased from American Type Culture Collection (ATCC).

### 3.2. Isolation of Streptomyces Strain 

Soil samples were collected from different regions of Columbia, Missouri, USA at a depth of 15–20 cm into sterile containers. Five grams of each soil sample were diluted in 50 mL of International *Streptomyces* Project (ISP2) medium [62] and the inoculated medium was placed in a 30 °C shaker at 100 rpm for 60 min. Ten mL of three different dilutions (10^−1^, 10^−2^, 10^−3^) were prepared in sterile saline solutions 0.85% (*w/v*) NaCl. Then, 100 μL of each dilution was cultured on ISP 2 solidified medium composed of the following in one liter: yeast 4 g, malt 10 g, glucose 4 g and agar 15 g and Glycerol–asparagine agar medium which is composed of the following in one liter (asparagine 1 g, glycerol 10 g, anhydrous K_2_HPO_4_ 1 g, 1 mL trace salt solution (0.1 g FeSO_4_·7H_2_O, 0.1 g MnCl_2_·4H_2_O, 0.1 g ZnSO_4_·7H_2_O and 100 mL H_2_O), 15 g agar). Nystatin 100 mg/L was added to each medium as an antifungal agent. Plates were incubated at 30 °C and monitored each 24 h for 14 days. Typical chalky *Streptomyces* colonies with earthy odor were selected and then streaked on ISP agar medium [63] and examined microscopically. The isolated *Streptomyces* species were preserved in the production medium (see Section 3.4) with glycerol at final concentration 20% (*v/v*) and kept at −20 °C.

### 3.3. Identification of the Streptomyces Strain 

A *Streptomyces* strain was identified on the basis of 16S rDNA gene sequencing where DNA was isolated from 1 mL of an overnight culture grown aerobically in production medium (see Section 3.4) with a Promega Wizard Genomic DNA purification kit according to the manufacturer’s instructions. For PCR amplification of the 16S rDNA gene, (Table 1) the following universal primers were used, FD1 and 1492R, and amplification was done with Herculase II fusion DNA polymerase. The amplified DNA product was purified with the Wizard PCR clean-up system (Promega Corp., Madison, WI, USA). For sequencing, we used the following primers FD1, 334F, 519F, 529R and 1099F and 1492R. The 16S rDNA (1471 nucleotides) sequence was compared to the sequences available in the National Center for Biotechnology Information (NCBI) database (5 March 2017). The phylogenetic analysis was performed with MEGA version 6 software [64].

### 3.4. Production Medium of EPS

EPS was produced in a production medium composed of the following in one liter (tryptone 5 g, yeast extract 5 g, glucose 10 g, anhydrous K_2_HPO_4_ 3 g, KH_2_PO_4_ 1 g, NaCl 3 g, MgSO_4_·7H_2_O 0.5 g, anhydrous CaCO_3_ 0.5 g) pH 7.0 [69]. Cells were removed by centrifugation at 17,136× *g* for 20 min, this was followed by precipitation of the EPS from cell-free culture medium with two volumes of cold ethanol 100% *v/v* and stored overnight at 4 °C. The precipitated EPS was then collected by centrifugation at 17,136× *g* for 20 min at 4 °C. The EPS was re-dissolved in distilled water and precipitated with two volumes of cold ethanol 100% *v/v*, this step was repeated three times. The white EPS precipitate was collected by centrifugation and lyophilized for further analysis.

### 3.5. EPS Spectral Analyses

For the detection of the functional groups of MOE6-EPS, infrared spectrum for MOE6-EPS was recorded with Fourier transform infrared (FTIR) spectroscopy (Thermo Nicolet, NY, USA). An EPS sample of two milligrams was ground with 200 mg of dry KBr powder and then pressed into a pellet which was used for FTIR analysis in the frequency range of 4000–400 cm^−1^.

For UV visible analysis, (250 μg/mL) MOE6-EPS was dissolved in distilled water and then detected with a UV-visible spectrophotometer (Cary 50 Bio UV-Visible) at wavelengths ranging from 200–400 nm.

The NMR experiments were performed on a Bruker AVIII HD 600 MHz NMR spectrometer equipped with a triple resonance cryogenic probe. About 5 mg of MOE6-EPS sample was dissolved in 0.75 mL of D_2_O and placed in a 5 mm NMR tube. The temperature of the sample was 25 °C for all the NMR experiments. Chemical shift axis was calibrated with respect to the residual solvent proton at 4.78 PPM. Proton NMR was acquired with and without water suppression.

The MALDI-TOF was done by dissolving 3 mg MOE6-EPS/mL H_2_O. Serial dilutions (1:1, 1:5, 1:10, and 1:20 *v*/*v*) of the sample were made into 2,5-dihyroxybenzoic acid (DHB) or a 9:1 mix of DHB and 2-hydroxy-5-methoxybenzoic acid (superDHB), both at 10 mg/mL in a solution of 60% (*v/v*) acetonitrile, 1% (*v/v*) formic acid in H_2_O. An aliquot (1 μL) was spotted onto a teflon-coated stainless steel MALDI plate and allowed to co-crystalize at room temperature. Once the samples were dry, the plate was loaded into the instrument, and high voltage turned on for 20 min prior to data acquisition (to stabilize flight-tube voltages). Adding salt to the DHB matrix can help with carbohydrate ionization (by the formation of Na-adducts) [70]. Addition of 0.5 uL of 0.5M NaCl to the spotted 1 μL sample (prior to complete drying) did improve signal to noise slightly for low-mass ions.

The Voyager DE-PRO instrument was operated in linear positive-ion mode (25 kV acceleration voltage, 300 ns delayed extraction, 7500 to 20,000 mass range) and positive-ion reflector mode for low mass (600–4000 *m*/*z*, 25 kV acceleration, 150 ns delayed extraction). Spectra were acquired at a laser intensity of 2300 (linear mode) and 2000 (reflector mode). Each spectrum consisted of 500 shots per acquisition. Each sample was calibrated against a close external spot containing intact proteins (AB Sciex Cal mix 3). The average mass error for these ions was ~2.5 Da. Masses of compounds should be accurate to within 5Da with this close external calibration.

### 3.6. Sugar Analysis

For detection of the monosaccharide composition of the MOE6-EPS, 10 mg of EPS was hydrolyzed by boiling with one mL of 2 M trifluoroacetic acid (TFA) at 110 °C for 6 h. Excess TFA was removed by lyophilization. The hydrolysis product was detected by HPLC on an Aminex column HPX-87N with the following dimensions: 300 mm × 7.8 mm, with deionized water as mobile phase and a flow rate of 0.5 mL/ min at 80 °C monitored with a refractive index detector.

### 3.7. Neutral Sugar, Uronic Acid and Protein Analyses

The neutral sugar contents in MOE6-EPS were determined by the phenol-sulfuric acid method with glucose as the standard at 490 nm [21]. Proteins were determined by Bradford assay with bovine serum albumin as a standard [23]. The uronic acid contents were determined by m-hydroxybiphenyl at 525 nm with glucuronic acid as the standard [22].

### 3.8. Antioxidant Activity of EPS

#### 3.8.1. DPPH Radical-Scavenging Assay

The DPPH (2,2-diphenyl-1-picrylhydrazyl) free radical-scavenging activity of the produced EPS was measured according to Yin et al., 2010 [71]. One mL of 0.2 mM DPPH in ethanol 100% *v/v* was mixed vigorously with one mL of EPS sample of different concentrations (0.5, 1, 1.5, 2, 3, 4 mg/mL). Then the mixture was incubated in the dark for 30 min. The absorbance of triplicate samples was measured at 517 nm and the absorbance compared to that of the ascorbic acid in the following concentrations (0.5, 1, 1.5, 2, 3, 4 mg/mL) was used as a positive control.

The DPPH scavenging percentage activity was calculated as follows [70]:

Scavenging ability % = [1 − (A_s_ − A_b_)/A_c_] × 100 (1)

where A_s_ is the absorbance of EPS sample with DPPH, A_b_ is the absorbance of the blank which is the absorbance of EPS without DPPH, A_c_ is the absorbance of the negative control which is the absorbance of DPPH alone without EPS.

#### 3.8.2. Hydroxyl Free Radical-Scavenging Assay

Hydroxyl free radical-scavenging activity was estimated following the method reported by Yin et al., 2010 [71] with some modifications. Where 2.0 mL of phosphate buffered saline (PBS, 20 mM, pH 7.4), 1.0 mL of 2.5 mM 1, 10-phenanthroline, 1.0 mL of 2.5 mM FeSO_4_ and 1 mL of 20 mM H_2_O_2_ were mixed. This was followed by the addition of one mL of various concentrations of the MOE6-EPS sample (0.5, 1, 1.5, 2, 3, 4 mg/mL) and then the whole mixture was incubated for one hour at 37 °C. The mixture absorbance was measured at 536 nm and the hydroxyl radical scavenging activity of the EPS was calculated as follow:

Scavenging ability % = [(A_s_ − A_c_)/(A_o_ − A_c_) × 100] (2)

where A_s_ is the absorbance of the mixture with different concentrations of EPS sample, A_c_ is the absorbance of the control without EPS sample, and A_o_ is the absorbance of the mixture in the absence of both H_2_O_2_ and the EPS sample. Ascorbic acid (0.25, 0.5, 1, 1.5, 2, 3, 4 mg/mL) was used as a positive control.

#### 3.8.3. Ferrous Ion Chelating Assay

The Fe^2+^ chelating assay was measured following the method of Qiao et al., 2009 [36]. Briefly, 1.0 mL of MOE6-EPS solution (0.25, 0.5, 1, 1.5, 2, 3, 4 mg/mL) was mixed well with 0.05 mL of a 2 mM ferrous chloride (FeCl_2_·4H_2_O) solution, which was followed by addition of 0.2 mL of a 5 mM ferrozine solution and then the final volume was adjusted to 4 mL with water. The mixture was shaken and incubated for 10 min at room temperature, after which, the absorbance of the mixture was measured at 562 nm with deionized water and EDTA-Na in same concentration of EPS as the blank and a positive control, respectively. The ferrous ion-chelating ability was calculated as follows:

Fe^2+^ chelating % = [(A_b_ − (A_s_ − A_o_)/A_b_)] × 100 (3)

where A_s_ is the absorbance of EPS solution, A_b_ is the absorbance of the blank, A_o_ is the absorbance of the same concentration of sample with water instead of FeCl_2_.

### 3.9. Effect of MOE6-EPS on Breast Cancer Cells

#### 3.9.1. Scratch-Wound Assay 

Cancer cells (4T1 mouse breast cancer cells) were seeded in a 6-well plate for 48 h in Dulbecco’s Modified Eagle’s medium (DMEM). When the cells formed a confluent layer, the scratch-wounds were made with sterile micropipette tips in each well and fresh DMEM containing 1% *v/v* fetal bovine serum was added with and without MOE6-EPS at different concentrations of 0, 1, 2 and 4 mg/mL. The healing of the scratch was monitored for 24 h by real time imaging on a Nikon TI-E inverted microscope with a humidified incubation chamber maintained at 37 °C, 95% air and 5% CO_2_. The cell migration rate was measured as the ratio of migration distance to a total distance of the wound gap:

Migration % = ((original scratch width − final scratch width)/original scratch width) × 100.(4)

#### 3.9.2. MTT Cell Proliferation Assay

The inhibition of mammalian cell viability by the produced EPS was examined by measuring the cell proliferation rate of tumor cells with a 3-(4,5-dimethylthiazol-2-yl)-2,5-diphenyltetrazolium bromide (MTT) assay described by Mosmann [72] where the cancer cells (MDA-MB 231 human breast cancer cell line) were seeded in a 24-well plate at a density of 3 × 10^4^ cells/well. After 24 h, fresh DMEM was added to the cells with increasing concentrations of MOE6-EPS (0, 2, 4, 8 and 16 mg/mL) at 37 °C and 5% CO_2_ in air. The growth of the cells was monitored for 96 h and then the cell viability was assessed with the Vybrant MTT assay kit (Thermofisher, Waltham, MA, USA) according to the manufacturer’s suggested protocol. Absorbance (570 nm) of the formazan produced from the reduction of the tetrazolium dye was measured on an Enspire 2300 Multilabel Reader (Perkin Elmer, Waltham, MA, USA). The MTT assay was performed three times with three replicates conducted for each experiment.

### 3.10. Statistical Analyses

Data represent means ± SE of three experiments. Statistical significance was defined as *p* < 0.05 and was calculated by Student’s *t*-test, with Graph Pad Prism software (GraphPad Software, Inc., San Diego, CA, USA).

## 4. Conclusions

In conclusion, in this study we report for the first time that the *Streptomyces* sp. MOE6, which was isolated from a soil sample, produced an EPS composed mainly of glucose, mannose and glucuronic acid. The partially purified EPS showed antioxidant activities by scavenging DPPH and hydroxyl radicals. Moreover, it exhibited metal chelation activity. Additionally, the EPS reduced the migration and the proliferation of mouse (4T1) and human breast cancer cell lines (MDA-MB 231), respectively. These results were interpreted to mean that the EPS from *Streptomyces* sp. MOE6 might be a candidate for the development of novel antioxidant and mammalian cell inhibitor drugs from natural sources.

## Figures and Tables

**Table 1:** Nucleotide sequences for PCR amplification and sequencing primers.

Primers	Nucleotide Sequences (5′ to 3′)	References
FD1	AGAGTTTGATCCTGGCTCAG	[65]
E334F	CCAGACTCCTACGGGAGGCAGC	[66]
519F	CAGCAGCCGCGGTAA	[67]
1099F	GCAACGAGCGCAACCC	[67]
529R	CGCGGCTGCTGGCAC	[67]
1492R	GGTTACCTTGTTACGACTT	[68]
